# Changes in Oral Papilloma Virus Infections Over Six Months in People Living with HIV

**DOI:** 10.21203/rs.3.rs-6495161/v1

**Published:** 2025-04-23

**Authors:** Ian G. Munabi, Adriane. Kamulegeya, David P. Kateete, Fred. Semitala, Samuel. Kalungi, Jennifer E. Cameron, Lauren L. Patton, Kimon. Divaris, William Buwembo

**Affiliations:** Department of Dentistry, School of Dentistry, Makerere University College of Health Sciences, Kampala, Uganda; Department of Oral and Maxillofacial surgery, School of Dentistry, Makerere University College of Health Sciences, Kampala, Uganda; Department of Immunology, School of Biomedical Sciences, Makerere University College of Health Sciences, Kampala, Uganda; Department of Medicine, School of Medicine, Makerere University College of Health Sciences, Kampala, Uganda; Department of Pathology, Mulago National Referral and Teaching Hospital, Kampala, Uganda of Medicine, School of Medicine, Makerere University College of Health Sciences, Kampala, Uganda; Louisiana State University Health Sciences Center: New Orleans, LA, USA; Department of Craniofacial and Surgical Care, Adams School of Dentistry, University of North Carolina Chapel Hill, North Carolina, USA. f Pediatric Dentistry and Dental Public Health, Adams School of Dentistry, University of North Carolina Chapel Hill, North Carolina, USA.; Department of Pediatric Dentistry and Dental Public Health, Adams School of Dentistry, University of North Carolina Chapel Hill, North Carolina, USA., Department of Pediatric Dentistry and Dental Public Health, Adams School of Dentistry, University of North Carolina Chapel Hill, North Carolina, USA.Gillings School of Global Public Health, University of North Carolina Chapel Hill, North Carolina, USA,; Department of Anatomy, School of Biomedical Sciences, Makerere University College of Health Sciences, Kampala, Uganda

**Keywords:** PLHIV, PV clearance, PV persistence, Papilloma viruses’ infections

## Abstract

There is a paucity of data on changes in oral papilloma virus (PV) infection in people living with HIV (PLHIV) especially in low resource settings. The objective of this study was to determine the changes in oral PV infections in PLHIV from a low resource setting over a six-month follow-up period. This was a cohort study in which data was derived from a sub-sample of a parent study that examined oral human papilloma viruses, microbiota, and cancer in PLWHIV. This as a six-month follow up and a 2 mls saliva sample was collected from 541 participants on both visits. The saliva sample was used for DNA extraction, PV screening and typing using PCR methods. The DNA was subjected to Nanopore PV sequencing and subsequently analyzed using the phyloseq object, followed by a series of comparisons using the Phyloseq and Vegan packages in R to generate the alpha and beta diversity indices of the sequencing data from the sampled participants PV OTUs at the two visits. We found that 60% of participants had no detectable PVs at six-month follow-up, with a significant clearance rate of 84.47%. Oncogenic PVs were less likely to be detected as new infections compared to non-oncogenic PVs (Rate Ratio (RR) 0.42, 95% CI 0.31 to 0.56, P < 0.01). Oncogenic PV types were more likely cleared than non-oncogenic strains (RR 1.16, 95% CI 1.03 to 1.31, P = 0.02), but persistence rates did not significantly differ. This study highlights important trends in the natural course of oral PV infections, demonstrating that while most infections clear over time, there are distinct differences in the behavior of oncogenic versus non-oncogenic strains. These findings have important implications for the understanding of PV epidemiology and may guide future preventive and therapeutic strategies, particularly in the context of Human PV-related cancer prevention.

## Introduction

Infections due to Papilloma viruses (PVs) are globally endemic and recognized as a type of sexually transmitted infection by the World Health Organization (WHO) [1–3]. It is estimated that approximately 11.4% of the worldwide population is affected by PVs with the highest prevalence in Sub-Saharan Africa [3, 4]. PV infections are typically self-limiting being cleared by the host’s immunity in most healthy individuals[5], although in some cases the infections may persist [6, 7]. In young people, there is an increased risk of infection due to higher sexual activity that may lead to quick re-infection prolonging the exposure to PVs [7]. This sexual activity may also introduce oncogenic genital PV types into the oral cavity [7]. HIV infection promotes PV infection through the HIV-tat protein which transactivates the PV long control region, which in turn increases the expression of the E6 and E7 early oncogenes [8–10]. The augmentation effect of HIV-tat extends to the PV E6 and E7 oncoproteins and leads to increased production of L1 which is responsible for increased infectivity of PV [11]. HIV infection also reduces PV infected cell elimination through the selective destruction of CD4 + lymphocytes and impairing the dendritic cell activation and activity of CD8 + lymphocytes [12, 13]. The risks of PV acquisition and related complications do not appear to decline with antiretroviral treatment [14, 15].

PV infection remains one of the major factors implicated in the development of squamous cell carcinoma of the cervix, vagina, vulva, penis, larynx, head, and neck [16, 17]. A typical infection duration starts with PV accessing the epithelial basement membrane through breaks in the epithelium following micro-trauma, erosion, or inflammation, to which the PV particles attach using the L1 protein and later infect the basal epithelial cells [18, 19]. Over the course of time, the PV takes over the cellular process to make several copies of itself that are eventually shed as the infected epithelial cell reaches the epithelial surface [19]. This cycle is repeated for as long as there are infected basal cells in the epithelium from basal cell division or access points following trauma [19]. This means that any condition that leads to increased exposure of the basement membrane like periodontitis [20, 21], that is commonly seen in patients with HIV [22], may lead to increased and/or longer duration of PV infection or exposure. Risk factors for oral PV infection include oral sexual contact [23], age [23], smoking [23–25], tonsillectomy and years since HIV diagnosis [26], inflammatory states that include periodontitis [20, 21], low CD4 + cell count in people living with HIV (PLHIV) [12, 27], and concurrent oral and genital PV infections in women [28]. PV infections are an important driver of malignancy in people living with HIV [29].

It is important to note that most PV infections last for a relatively short time ranging from less than 6 months to about two years. In some people the infections last longer and may eventually manifest as cancer. In mixed PV infections, that is, where there is more than one type of PV causing disease, high PV viral loads can be used as biomarkers for HIV disease progression [30]. Also, PLHIV with lower CD4 + cell counts are more likely to present with mixed PV infections compared to HIV negative individuals [31]. In HIV negative people, infection with multiple PV types increases the HIV acquisition risk by 20% for each additional PV type detected [32]. Mixed PV infections, especially those involving the oncogenic PV genotypes, may persist longer than non-mixed PV infections [24, 33]. In addition to behavioral factors like having multiple sexual partners and smoking [25], interactions between viruses during multiple PVs infections, may in part also explain the high prevalence of PV DNA in PLHIV [7, 27, 34–37]. There is a paucity of data on the changes in oral PV infections in PLHIV in our low resource setting. In this manuscript we set out to determine the oral PVs changes in PLHIV from a low resource setting over a six-month follow-up period.

## Methods

As has been described elsewhere [38], the participants in this cohort study, were at baseline drawn consecutively from Makerere University Joint AIDS Program clinic (MJAP), situated in Kampala Uganda which serves a total of 18,000 PLHIV drawn from both the urban and peri-urban populations of south-central part of Uganda in East Africa [39]. The data in this report were generated from a sub-sample of the parent case control study that examined oral human PV, microbiota and cancer in PLHIV [40]. In the parent study, participants who were registered patients in the clinic, above the age of 18-years and had provided informed consent, were consecutively recruited over a 6-month period to attain a target sample size of 4,600. The sample size for this cohort sub-study was obtained using the online calculator for Sample size for Survival analysis [41], for the following assumptions: 0.5 as the proportion of participants in both the exposed to the known oncogenic types of PV (16, 18, 31, 33, 35, 39, 45, 51, 52, 56, 58 and 59 [42, 43]) compared to the other non-oncogenic types of PV; Relative hazard ratio of 0.44 [44], Median survival time with persistence 2.4 [44], censored rate of 0.35 [44], and a maximum of one follow-up visit time unit of six-months; Power 0.95 and significance 0.05. This resulted in an estimated sample size of 492 PLHIV for a total of 77 events (clearance of the previously identified PV infection as evidenced by a negative PV test at next review visit). This was increased by a 10% allowance for loss to follow-up, errors and missing data and rounded up to 542 participants for follow-up. Inclusion criteria: (a) having been seen as part of the baseline study and found being positive for any one of the known PV types, (b) absence of lesions on oral examination at the baseline visit and (c) consent to participate in the follow-up study. Sampling: The participants who qualified for inclusion were selected randomly using computer generated numbers based on MJAP clinic patient identification numbers.

### Study procedure:

Participants who qualified were contacted by phone and requested to return for a repeat assessment 6 months after the initial examination. All participants who agreed to come for the follow-up examination were reconsented on the day of their visit. As was done at the baseline visit, each reconsented participant was asked to provide a sample of saliva for five minutes. After five minutes, participants were asked to top up their samples to 5mls. These saliva samples were immediately placed on ice for transfer to the laboratory within two hours of collection. In the lab, each sample was aliquoted into two 2ml batches that were both centrifuged at 4000 revolutions per minute for four minutes. The supernatant from each of these samples was discarded and one of the remaining pellets was immediately placed in Cell lysis solution of the DNA extraction Kit (D4069, Zymoresearch, CA, USA). A second pellet was placed in a cryovial at minus 20 degrees Celsius for later transfer to minus 80 degrees Celsius for long term storage. The remaining 1ml sample was stored immediately at minus 20 degrees Celsius and later transferred to long term storage at minus 80 degrees. The molecular techniques in this manuscript have previously been used by our research group[45].

### DNA Extraction, PV screening and typing:

DNA extracted from the pellets was done using Quick-DNA Mini prep Plus Kit (D4069, Zymoresearch, CA, USA), with overnight protein kinase digestion, as per the manufacturer’s instructions. The extracted DNA from each sample was quantified using a Nanodrop colorimeter (Thermofisher scientific, MA, USA) following the manufacturer’s instructions. This was followed by subjecting 10-micro-liters of each sample to PV confirmatory screening using the forward FAP 59 and reverse FAP 64 primers [37], that were each respectively designed with an additional forward or reverse nanopore tag. DreamTaq DNA polymerase under the following assay conditions: 5 min at 94 °C, 40 cycles (denaturation 94 °C/30 s, annealing 52 °C/45 s, and extension 72 °C 1 min) followed by a final extension step at 72 °C for 7 min. The 490bp band PV positive PCR products were visualized using 2% agarose gel with ethidium bromide. Samples without any band from the FAP primers were subjected to an additional confirmatory PCR screening for 150bp bands using the double nested PGmy9/11 and Gp+5/+6 primers and protocol [46]. All the HPV positive samples in the study pool were subjected to further PCR based typing using the Sotlar method [47].

### Nanopore PV sequencing:

Sequencing was carried out on 72/541 samples that had been identified as having remained PV positive at both time points, using the SQK-LSK114 sequencing kit as per manufacturer’s instructions using the protocol for nanopore tagged amplicons [48], with the following modifications. After the above FAP and or GP5+ PCR, the amplicons were cleaned up with Clean NGS beads (Coenecoop 75, 2741 PH Waddinxveen, Netherlands) with the ratio of 1:2 of the amplicons to the beads as per the manufacturer’s instructions. A second bead clean of 1:1 was performed using the supernatant and the beads. The supernatant was removed and the DNA bound to the beads was washed twice with 800μl of freshly prepared 70% ethanol, without disturbing the beads. The beads and DNA were re-suspended in 10 μl of Nuclease free water and incubated at room temperature for 10 min. The sample containing DNA and the beads was pelleted on a magnetic rack and the supernatant containing DNA was collected. The cleaned amplicons were quantified using a Qubit^™^ 4 Fluorometer (Themo Fisher Scientific, City, Singapore). The purified PCR products were used for Nanopore PCR barcoding using the EXP-PBC096 PCR barcoding expansion pack (Oxford Nanopore Technologies plc, Oxford, UK) under the following conditions 37-degrees Celsius for 20 min and 87-degrees Celsius for 20 min. The barcoded PCR products were purified with Clean Next Generation Sequencing (NGS) beads in a ratio of 1:0.4 for the sample to beads before pooling in equimolar volumes for Ligation using the SQK-LSK114-XL kit (Oxford Nanopore Technologies plc, Oxford, UK) as per the manufacturer’s instructions. The final DNA library was quantified using both Nano drop One (Themo Scientific, USA) and Qubit^™^ 4 Fluorometer (Themo Fisher Scientific, City, Singapore). The quantified DNA library not exceeding 10-fetomolar based on the Qubit^™^ 4 Fluorometer (Themo Fisher Scientific, City, Singapore) measurement for each run, was loaded onto the MinION MIN-101B device running a flongle Flow Cell FLO-FLG114 (Oxford Nanopore Technologies plc, Oxford, UK) with the until end-of-life setting.

### Processing of sequencing data:

The data for 72 participants, corresponding to their baseline and follow-up visits, were initially saved as POD5 files during sequencing. Using a python 3.12 jupyter notebook and a series of bash commands, the sequencer-generated POD5 files were converted first to bam files using bash commands for the GPU version of the manufacturer’s demultiplexing software, Dorado version 8.1 running on a Nvidia GPU 3070, AUSUS Tuf Dash F15 i7 64GB ddr5 ram laptop. The highest quality ONT dorado v5 SUP models to ensure that the reads obtained are of the best quality. Sequencing reads with a minimum quality Phred score of 5, were retained in bam files for subsequent analysis. The data in the bam file were converted to the fasta format using SAMtools [49], and later to blast output summary text files of the aligned reads using the whole genome reference sequences for all known PVs from Pave database (www.pave.niaid.nih.gov (22 December 2024)), for importing into R. At the blast step, only reads longer than 100 bases after trimming the nanopore sequencing adapters, primers and tags, were retained for the blasting against the reference genomes, using the highest settings (-c50 -s) of the blastn based NanoBLASTer [50]. Use of whole genome PV reference data allowed the blast step to capture data both the amplified segments (L1, E6 and E7) and any other regions of the PV virus in the test solutions. Downstream analysis in the R statistical computing environment made use of the data.table (version 1.16.4) related R packages for both the initial data wrangling and later generation of summaries from the data using the identified PV related reads.

### Analysis of the data:

Data analysis: The data were downloaded as excel sheets from redcap and imported into the R version 4.4.2 statistical computing environment running on windows for data wrangling to merge and label all the relevant pieces of data before the production of summaries. Descriptive data summaries were based on counts and frequencies that were followed by a series of multilevel regression analyses using the glmer function of the Lme4 package [51], with a Poisson distribution to compute the rate ratios of the PV infection count outcomes controlling for individual participants. For the sequencing PV data, text or excel file summaries were imported into R and reorganized into a phyloseq object using in-house scripts. Analysis of the sequencing data in the phyloseq object involved recording the total number of segments corresponding to viral genomes or OTUs followed by a series of comparisons using the Phyloseq [33] and Vegan [34] packages in R to generate the alpha and beta diversity indices of the sequencing data from the sampled participants PV OTUs at the two visits. The R code and related datasets used in this sub-analysis have been included as part of the supplementary data supporting this report.

#### Ethical considerations:

This study has been approved by Ethics Committee of Makerere University School of Medicine Research and Ethics committee (SOMREC) and the Uganda National Council of Science and technology ((UNCST). The ethics approval numbers are REC REF 2022–451 and HS2541ES respectively. The ethics approval date was November 22, 2022. All procedures followed were in accordance with the ethical standards of the committee responsible for human experimentation (institutional and national) and with the Helsinki Declaration. Informed consent was obtained from all participants, in writing and dated, before enrolment in the study.

## Results

**Figure 1:**
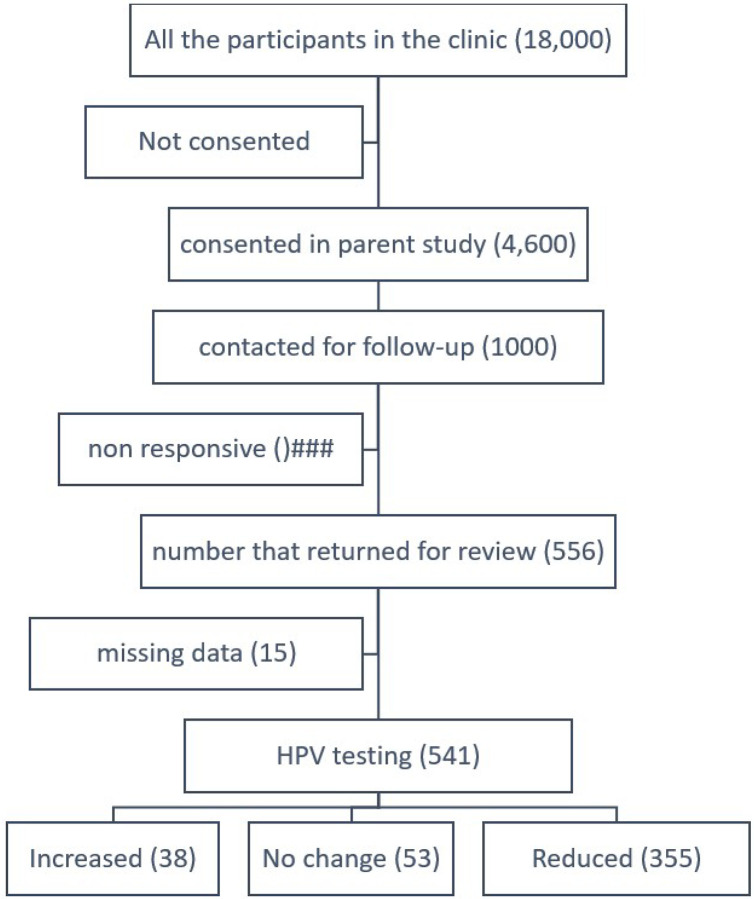
Participant flow diagram

As shown in the participant flow diagram (see Figure 1), a total of 556 participants presented for their agreed upon follow-up appointment. Fifteen of them had missing data and were removed from further analysis, leaving a total of 541 participants. Most participants (75%) were female and 45 years (SD=10) of age. The average follow-up time for all the participants that returned for examination was 5.9 (SD 0.53) months. As summarized in Table 1, most (60%) participants had no detectable infection by the time of the follow-up visit. Also, in Table 1, note the overall reduction in the combinations of PV types detected in participants’ samples for the two visits.

### Changes in detected PV types at the two participant contact times

Considering individual PV infection types, there were 1365 (85%) instances of the previously detected PV type infections that were cleared at the second follow-up contact. There were also 196 (12%) instances of new or incident PV type infections and another 55 (3%) instances of PV type infections that were present at both the baseline and follow-up time points (persistent). Table 2 provides a summary of the changes in PV infections for each of the tested PVs for each of the participants comparing presence at the two time points. Oncogenic PVs were less likely to be detected as new infections compared to non-oncogenic PVs. This was significant (Rate Ratio (RR) 0.42, 95% CI 0.31 to 0.56, P <0.01), and remain unchanged on controlling for gender (Female = RR 1.34, 95% CI 0.86 to 2.07, P= 0.19), unit increase in age (RR = 0.99, 95% CI 0.98 to 1.01, P= 0.41) and each additional month of follow-up (RR 1.16, 95% CI 0.80 to 1.67, P= 0.44). Oncogenic PVs were more likely to be cleared compared to the non-oncogenic PVs. This too was significant (RR 1.16, 95% CI 1.03 to 1.31, P =0.02). This remained unchanged on adjusting for gender (Female = RR 0.96, 95% CI 0.85 to 1.09, P= 0.56), unit increase in age (RR = 1.00, 95% CI 1.00 to 1.01, P= 0.65) and each additional month of follow-up (RR 0.99, 95% CI 0.87 to 1.13, P= 0.93). Oncogenic PVs were more likely to be detected as persistent PV infection compared to non-oncogenic PVs. This was not significant (RR 1.02, 95% CI 0.57 to 1.83, P= 0.95).

### Results of sequencing samples with persistent PVs

Sequencing generated 2,791,802 reads of which 405,864 (14.54%) mapped onto one of the known PV genomes. There were only 207,566 of the 405,864 (51.14%) PV reads that had barcodes for linking to the sample data. There were 95,384/207,566 (45.95%) PV reads that could be linked to 72/541 followed-up participants sample identification numbers. A total of 214 PV OTUs or genomes were identified. Table 3, which provides a summary of both the alpha diversity indices, shows there were about the same PV genomes at the first visit (191) compared to the second visit (181). All the other alpha diversity measures were within the same range for the two visits. In the case of similarity indices, the Bray Curtis index was 0.17 while the Jaccard index was 0.45 indicating low similarity of organisms between the first and second visit. There was no association between the distribution of the PVs with regards to the participants first or second visit (F-statistic = 1.40, P = 0.18) age (F-statistic = 0.62, P = 0.65) or gender (F-statistic = 0.82, P = 0.46). Table 4 provides a list of the PVs with the highest number of reads for both the baseline and follow-up visit.

## Discussion

We set out to determine changes in PV related oral infections affecting PLHIV over a six-month follow-up period. Most of the previously PV positive participants had no detectable PVs after the follow-up period. Among those that remained positive there was a general reduction in the number of reads and combinations of PVs found in each participant. The analysis of changes in detected PV types between two contact points for participants reveals key insights into the dynamics of PV infections over time. These results indicate that most PV infections either cleared or did not persist over the follow-up period. This aligns with the natural history of PV infections that PV remains in the epithelial layer of cells until the previously infected basement membrane cells are exhausted, which is further supported by the fact that 85% of previously detected PV types were cleared by the second follow-up visit. This highlights the potential for spontaneous resolution of PV infections, particularly those associated with non-oncogenic strains and is in line with what has been reported in literature with up to 80% clearance within a 6.5-to-18-month period [5].

We found that the oncogenic PV infections were more likely to be cleared compared to non-oncogenic infections. This finding is consistent with previous research indicating that the immune response to oncogenic PV types, such as Human PV 16 and Human PV 18, may be more pronounced. However, their clearance at 6 months is low compared to the other oncogenic PVs [52]. In our study we identified more non-oncogenic PVs compared to oncogenic PVs which may explain the high clearance within the six-month follow-up period. This is in line with the findings from Balkans et al [52]. It is worth noting that some other studies that used modeling did not show any difference in clearance between the different Human PVs based on oncogenicity [53]. The higher clearance rate of oncogenic PV in our study is not surprising since HIV serostatus has not been reported to affect one’s ability to clear high-risk PVs. However, some studies have reported a negative effect of HIV sero-status on clearance [54], hence more research needs to be done for a better understanding of HIV infection’s impact on PV persistence [55].

Interestingly, the likelihood of persistence did not differ significantly between oncogenic and non-oncogenic PV types. From our data we found that only 3.4% of the infections were persistent across the two time-points. Persistence of PV in the oral cavity can have serious consequences, ranging from an increased risk of oral and oropharyngeal cancers to chronic inflammation and the development of other complications such as oral warts and condylomas [56–58]. Preventative measures, including vaccination against high-risk PV strains and early detection of PV-related oral lesions, are essential in minimizing these risks. Additionally, promoting a healthy lifestyle, avoiding smoking, and encouraging regular dental and medical check-ups can help reduce the chances of persistent PV infection in the oral cavity. Thus, finding no difference in the likelihood of persistence suggests that while the immune system may clear oncogenic PV infections more effectively and those infections that persist could have similar characteristics to non-oncogenic strains in terms of persistence. This could indicate that, in some of our participants, there are unique host factors that favor the establishment of persistent PV infections. These factors are separate from the above-mentioned general immune response. This implies that persistence of these PV infections is influenced by factors beyond viral oncogenic potential. Both the viral ability to evade the immune system and the host factors such as age and other behavior (smoking, contraceptive use and number of sexual partners) affect clearance [59].

From our data, we found that a subset of infections were either incident (12%) across the two time points. The presence of incident PV infections underscores the continued risk of acquiring new infections over time, despite prior exposure. This may be because each PV infection leads to the production of genotype-specific antibodies which may not provide protection against other PVs [60]. This failure to protect against other PVs has also been reported when it comes to vaccines. Of note is the statistically significant finding that oncogenic PV types were less likely to appear as new infections, a trend that contrasts with non-oncogenic PV types, suggesting that oncogenic PV types may exhibit a lower rate of acquisition compared to non-oncogenic PV types. This could be due to the immune system’s more robust response to oncogenic strains or differences in the biology of these viruses that make them less likely to be transmitted or because in this setting they are not as common to acquire as the non-oncogenic types.

Overall, the results presented here emphasize the variability in the persistence, acquisition, and clearance of PV infections, with notable differences between oncogenic and non-oncogenic types. These findings are consistent with the broader understanding of PV infection dynamics, wherein high-risk strains, such as Human PV 16 and Human PV 18, are less likely to be cleared and may persist for extended periods, potentially leading to complications like cancer [54, 55]. In contrast, low-risk strains typically clear more rapidly, reducing the overall burden of disease. Furthermore, the results suggest that age, gender, and duration of follow-up had limited impact on the detection and clearance of PV infections in this cohort. This indicates that these factors may not be as influential in the short-term dynamics of PV infections, although they could play a role over longer durations or in different populations as reported by other studies [54, 61]. The continued monitoring of this cohort and others could provide further insights into the long-term trajectories of PV infections, particularly regarding the persistence of oncogenic strains.

### Limitations

While the study provides valuable insights into the dynamics of PV infection over time, including differences in both the persistence and clearance of oncogenic and non-oncogenic types, it faces several limitations. These include potential biases due to participant selection, possible left censoring since we did not catch participants before the infection started, the short follow-up period, analysis restricted to only the known PV types in the pave database, and the lack of consideration of confounding factors such as sexual behavior, immune status, and vaccination status. Further studies with larger and more diverse populations, longer follow-up durations, and more detailed data on confounding factors are needed to deepen our understanding of PV infection dynamics in this setting.

## Conclusion

This study highlights important trends in the natural course of oral PV infections, demonstrating that while most infections clear over time, there are distinct differences in the behavior of oncogenic versus non-oncogenic strains. Oncogenic PV types are less likely to be newly acquired and more likely to be cleared, but persistence rates do not differ significantly between the two categories. These findings have important implications for the understanding of PV epidemiology and may guide future preventive and therapeutic strategies, particularly in the context of Human PV-related cancer prevention.

## Data Availability

The data that support the findings of this study are available on request from the corresponding author. The data are not publicly available due to privacy or ethical restrictions.
